# Reynolds Stress Model for Viscoelastic Drag-Reducing Flow Induced by Polymer Solution

**DOI:** 10.3390/polym11101659

**Published:** 2019-10-11

**Authors:** Yi Wang

**Affiliations:** National Engineering Laboratory for Pipeline Safety/MOE Key Laboratory of Petroleum Engineering/Beijing Key Laboratory of Urban Oil and Gas Distribution Technology, China University of Petroleum, Beijing 102249, China; wangyi1031@cup.edu.cn; Tel.: +86-1881-090-5760

**Keywords:** Reynolds stress model, polymer, turbulent model, drag reduction, DNS

## Abstract

Viscoelasticity drag-reducing flow by polymer solution can reduce pumping energy of pipe flow significantly. One of the simulation manners is direct numerical simulation (DNS). However, the computational time is too long to accept in engineering. Turbulent model is a powerful tool to solve engineering problems because of its fast computational ability. However, its precision is usually low. To solve this problem, we introduce DNS to provide accurate data to construct a high-precision turbulent model. A Reynolds stress model for viscoelastic polymer drag-reducing flow is established. The rheological behavior of the drag-reducing flow is described by the Giesekus constitutive Equation. Compared with the DNS data, mean velocity, mean conformation tensor, drag reduction, and stresses are predicted accurately in low Reynolds numbers and Weissenberg numbers but worsen as the two numbers increase. The computational time of the Reynolds stress model (RSM) is only 1/120,960 of DNS, showing the advantage of computational speed.

## 1. Introduction

Drag reduction (DR) phenomenon was first discovered by Toms [1]. He observed in his experiment that the addition of a long-chain polymer (polymethyl methacrylate) in monochlorobenzene dramatically reduced the turbulent skin friction by as high as 80%. The flow rate could be increased by the addition of the polymer at constant pressure gradient. Then, he reported these results at the First International Rheological Congress, so it is usually referred to as the “Toms Effect”. The polymers that can reduce skin friction were later called drag-reducing agents (DRAs). The energy-saving effect of DRAs attracts many applications. The first famous application for polymer drag reduction was its use in the 48 inch diameter 800 mile length Alaska pipeline, carrying crude oil from the North slope in Alaska to Valdez in the south of Alaska [2]. After injecting a concentrated solution of a high-molecular-weight polymer downstream of pumping stations at homogeneous concentrations as low as 1 ppm [3], crude throughput was increased by up to 30%. Polymer DRAs were also successfully applied in other crude oil pipelines such as Iraq-Turkey, Bass Strait in Australia, Mumbai Offshore [4], and North Sea Offshore [5], and in finished hydrocarbon product lines [6].

The drag-reducing flow induced by polymer solution usually appears viscoelastic. Direct numerical simulation (DNS) can simulate this kind of viscoelastic turbulent flow in high precision [7,8,9,10,11]. More recent progresses are as follow. Dubief et al. [12] investigated the energetics of turbulence by correlating the work done by polymers on the flow with turbulent structures. Polymers are found to store and to release energy to the flow in a well-organized manner. Graham [13] proposed a tentative unified description of rheological drag reduction based on his numerical observations. Thais et al. [14] found that the spectra of its cross-flow component in viscoelastic flows exhibit a significantly higher energy level at a large scale. Pereira et al. [15] studied the polymer–turbulence interactions from an energetic standpoint for a range of Weissenberg numbers and found a cyclic mechanism of energy exchange between the polymers and turbulence that drives the flow through an oscillatory behavior. They also addressed the numerical simulation of thermo-fluid characteristics of triangular jets [16]. However, DNS needs numerous storages of the computer because very dense mesh is required to resolve small eddies in turbulent drag-reducing flow, such that computational time is too long to be accepted in engineering. Turbulent model computes the turbulent flow very quickly. Compared with DNS, turbulent model for viscoelastic drag-reducing flows develops slowly. There were zero-Equation models established by Edwards et al. [17] and Azouz et al. [18]. One-Equation models and two-Equation models were established by Durst et al. [19], and Hassid and Poreh [20,21,22]. Cruz et al. [23] and Pinho et al. [24] considered elongation thickening of drag-reducing fluid based on Newtonian fluid turbulent flow and derived a new low Reynolds number *k-ε* model for polymer drag-reducing flow. Elongation thickening is a very important factor of drag reduction. Thus, Pinho’s work promoted the turbulent model greatly, but the precision is still not high enough due to the complexity of viscoelastic turbulent flow. Reynolds stress model (RSM) can simulate Newtonian turbulent flow in high precision, so that it has potential advantages to deal with the complex viscoelastic turbulent flow with polymer additives. If the RSM is established based on DNS data, the precision may be better.

In this paper, an RSM for viscoelastic drag-reducing flow is established based on DNS data. The goal is to find a new modeling way to solve drag-reducing flows in engineering with fast computation and good accuracy.

## 2. Governing Equations

### 2.1. Instantaneous Equations

Viscoelastic drag-reducing flow can be described by the following governing Equations [25].

(1) Continuity Equation:

(1) $$\frac{\partial u_{i}}{\partial x_{i}}=0$$

(2) Momentum Equation:

(2) $$\frac{\partial u_{i}}{\partial t}+u_{k}\frac{\partial u_{i}}{\partial x_{k}}=-\frac{1}{\rho }\frac{\partial p}{\partial x_{i}}+\frac{\mu }{\rho }\frac{\partial^{2}u_{i}}{\partial x_{k}^{2}}+\frac{1}{\lambda \rho }\frac{\partial c_{ik}}{\partial x_{k}}$$

**Figure 1:**
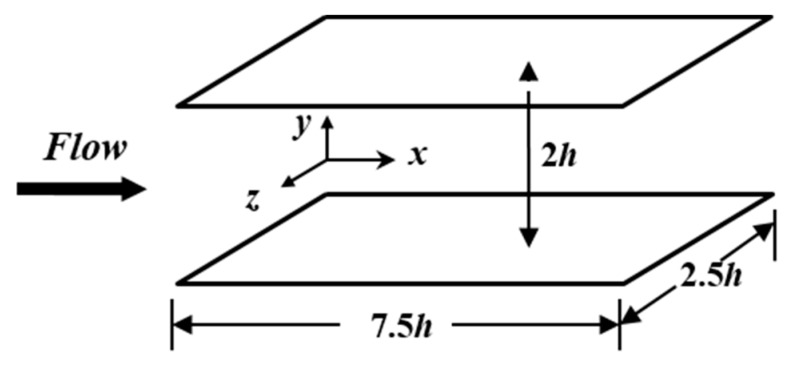
Computational domain.

(3) Giesekus constitutive Equation:

(3) $$\frac{\partial c_{ij}}{\partial t}+\frac{\partial u_{m}c_{ij}}{\partial x_{m}}-c_{mj}\frac{\partial u_{i}}{\partial x_{m}}-c_{im}\frac{\partial u_{j}}{\partial x_{m}}+\frac{1}{\lambda }\left[-\eta \delta_{ij}+c_{ij}+\frac{\alpha }{\eta }\left(c_{im}-\eta \delta_{im}\right)\left(c_{mj}-\eta \delta_{mj}\right)\right]=0$$

where ρ and λ are the density and the relaxation time of drag-reducing fluid respectively. α is the mobility factor, which determines the extensional viscosity. p is pressure. $u_{i}$ (*i* = *x*, *y*, *z*) are the velocity components in the *x*, *y*, *z* directions. $c_{ij}$ is the conformation tensor. μ is the zero-shear-rate viscosity of solvent. η is the zero-shear-rate viscosity of drag-reducing polymer. We choose the following dimensionless transformation: $x_{i}^{*}=x_{i}/h$, $t^{*}=t/\left(h/u_{\tau }\right)$, $u_{i}^{+}=u_{i}/u_{\tau }$, $p^{+}=p/\left(\rho u_{\tau }^{2}\right)$, $c_{ij}^{+}=c_{ij}/\eta$ and use the definition of Weissenberg number $We_{\tau }=\rho \lambda u_{\tau }^{2}/\left(\mu +\eta \right)$, the definition of frictional Reynolds number $Re_{\tau }=\rho u_{\tau }h/\left(\mu +\eta \right)$, and the ratio β=μ/(μ+η). $u_{\tau }$ is the frictional velocity ($u_{\tau }=\sqrt{\tau_{w}/\rho }$, $\tau_{w}$ is the wall shear stress). h is the half height of the channel, as shown in Figure 1. Using these definitions, Equations (1)–(3) can be transformed to be the following dimensionless Equations:

(4) Dimensionless continuity Equation:

(4) $$\frac{\partial u_{i}^{+}}{\partial x_{i}^{*}}=0$$

(5) Dimensionless momentum Equation:

(5) $$\frac{\partial u_{i}^{+}}{\partial t^{*}}+u_{k}^{+}\frac{\partial u_{i}^{+}}{\partial x_{k}^{*}}=-\frac{\partial p^{+}}{\partial x_{i}^{*}}+\frac{\beta }{Re_{\tau }}\frac{\partial^{2}u_{i}^{+}}{\partial x_{k}^{*2}}+\frac{1-\beta }{We_{\tau }}\frac{\partial c_{ik}^{+}}{\partial x_{k}^{*}}$$

(6) Dimensionless Giesekus constitutive Equation:

(6) $$\frac{\partial c_{ij}^{+}}{\partial t^{*}}+\frac{\partial u_{m}^{+}c_{ij}^{+}}{\partial x_{m}^{*}}=\frac{Re_{\tau }}{We_{\tau }}\left[\delta_{ij}-c_{ij}^{+}-\alpha \left(c_{im}^{+}-\delta_{im}\right)\left(c_{mj}^{+}-\delta_{mj}\right)\right]+\frac{\partial u_{i}^{+}}{\partial x_{m}^{*}}c_{mj}^{+}+\frac{\partial u_{j}^{+}}{\partial x_{m}^{*}}c_{mi}^{+}$$

### 2.2. Time-Average Equations

Reynolds stress model focuses on the time-average effect of turbulent flow. Thus, instantaneous variables in the above Equations can be considered as the summation of time-average variables and fluctuation variables, that is, $\varphi^{+}=\bar{\varphi^{+}}+\varphi^{{+}^{\prime }}$ ($\varphi^{+}$ represents instantaneous variables ($u_{i}^{+}$, $p^{+}$, $c_{ij}^{+}$ etc.); superscripts “—” and “’” represent time-average variables and fluctuation variables respectively). After this decomposition, time-average operations can be made for the instantaneous Equations to obtain the following time-average Equations:

(1) Time-average momentum Equations:

(7) $$\frac{\partial \bar{u_{i}^{+}}}{\partial t^{*}}+\bar{u_{k}^{+}}\frac{\partial \bar{u_{i}^{+}}}{\partial x_{k}^{*}}+\frac{\partial \bar{u_{i}^{{+}^{\prime }}u_{k}^{{+}^{\prime }}}}{\partial x_{k}^{*}}=-\frac{\partial \bar{p^{+}}}{\partial x_{i}^{*}}+\frac{\beta }{Re_{\tau }}\frac{\partial^{2}\bar{u_{i}^{+}}}{\partial x_{k}^{*2}}+\frac{1-\beta }{We_{\tau }}\frac{\partial \bar{c_{ik}^{+}}}{\partial x_{k}^{*}}$$

(2) Time-average constitutive Equations:

(8) $$\begin{array}{c} \frac{\partial \bar{c_{ij}^{+}}}{\partial t^{*}}+\frac{\partial \bar{u_{m}^{+}}\bar{c_{ij}^{+}}}{\partial x_{m}^{*}}+\underset{A_{ij}}{\underbrace{\frac{\partial \bar{u_{m}^{{+}^{\prime }}c_{ij}^{{+}^{\prime }}}}{\partial x_{m}^{*}}}}=\frac{Re_{\tau }}{We_{\tau }}\left[\delta_{ij}-\bar{c_{ij}^{+}}-\alpha \left(\bar{c_{im}^{+}}\bar{c_{mj}^{+}}-2\bar{c_{ij}^{+}}+\delta_{ij}\right)\right]-\underset{B_{ij}}{\underbrace{\alpha \frac{Re_{\tau }}{We_{\tau }}\bar{c_{im}^{{+}^{\prime }}c_{mj}^{{+}^{\prime }}}}}\\ +\frac{\partial \bar{u_{i}^{+}}}{\partial x_{m}^{*}}\bar{c_{mj}^{+}}+\frac{\partial \bar{u_{j}^{+}}}{\partial x_{m}^{*}}\bar{c_{mi}^{+}}+\underset{C_{ij}}{\underbrace{\bar{\frac{\partial u_{i}^{{+}^{\prime }}}{\partial x_{m}^{*}}c_{mj}^{{+}^{\prime }}+\frac{\partial u_{j}^{{+}^{\prime }}}{\partial x_{m}^{*}}c_{mi}^{{+}^{\prime }}}}} \end{array}$$

Equations (2)–(7) so that we can obtain the following fluctuation Equations.

(3) Fluctuation Equations:

(9) $$\frac{\partial u_{i}^{{+}^{\prime }}}{\partial t^{*}}+u_{k}^{{+}^{\prime }}\frac{\partial \bar{u_{i}^{+}}}{\partial x_{k}^{*}}+\bar{u_{k}^{+}}\frac{\partial u_{i}^{{+}^{\prime }}}{\partial x_{k}^{*}}=-\frac{\partial p^{{+}^{\prime }}}{\partial x_{i}^{*}}+\frac{\beta }{Re_{\tau }}\frac{\partial^{2}u_{i}^{{+}^{\prime }}}{\partial x_{k}^{*2}}+\frac{1-\beta }{We_{\tau }}\frac{\partial c_{ik}^{{+}^{\prime }}}{\partial x_{k}^{*}}-\frac{\partial }{\partial x_{k}^{*}}\left(u_{i}^{{+}^{\prime }}u_{k}^{{+}^{\prime }}-\bar{u_{i}^{{+}^{\prime }}u_{k}^{{+}^{\prime }}}\right)$$

Equation (9) can also be rewrote as:

(10) $$\frac{\partial u_{j}^{{+}^{\prime }}}{\partial t^{*}}+u_{k}^{{+}^{\prime }}\frac{\partial \bar{u_{j}^{+}}}{\partial x_{k}^{*}}+\bar{u_{k}^{+}}\frac{\partial u_{j}^{{+}^{\prime }}}{\partial x_{k}^{*}}=-\frac{\partial p^{{+}^{\prime }}}{\partial x_{j}^{*}}+\frac{\beta }{Re_{\tau }}\frac{\partial^{2}u_{j}^{{+}^{\prime }}}{\partial x_{k}^{*2}}+\frac{1-\beta }{We_{\tau }}\frac{\partial c_{jk}^{{+}^{\prime }}}{\partial x_{k}^{*}}-\frac{\partial }{\partial x_{k}^{*}}\left(u_{j}^{{+}^{\prime }}u_{k}^{{+}^{\prime }}-\bar{u_{j}^{{+}^{\prime }}u_{k}^{{+}^{\prime }}}\right)$$

Equation (9) × $u_{j}^{{+}^{\prime }}$ + Equation (10) × $u_{i}^{{+}^{\prime }}$ and do the time average, we can obtain the following Reynolds stress transport Equations.

(4) Reynolds stress transport Equations:

(11) $$\begin{array}{l} \frac{\partial \bar{u_{i}^{{+}^{\prime }}u_{j}^{{+}^{\prime }}}}{\partial t^{*}}+\bar{u_{k}^{+}}\frac{\partial \bar{u_{i}^{{+}^{\prime }}u_{j}^{{+}^{\prime }}}}{\partial x_{k}^{*}}=\underset{P_{ij}}{\underbrace{-\bar{u_{i}^{{+}^{\prime }}u_{k}^{{+}^{\prime }}}\frac{\partial \bar{u_{j}^{+}}}{\partial x_{k}^{*}}-\bar{u_{j}^{{+}^{\prime }}u_{k}^{{+}^{\prime }}}\frac{\partial \bar{u_{i}^{+}}}{\partial x_{k}^{*}}}}+\underset{\phi_{ij}}{\underbrace{\bar{p^{{+}^{\prime }}\left(\frac{\partial u_{i}^{{+}^{\prime }}}{\partial x_{j}^{*}}+\frac{\partial u_{j}^{{+}^{\prime }}}{\partial x_{i}^{*}}\right)}}}\\ \underset{T_{ij}}{\underbrace{-\frac{\partial }{\partial x_{k}^{*}}\left(\bar{p^{{+}^{\prime }}u_{i}^{{+}^{\prime }}}\delta_{jk}+\bar{p^{{+}^{\prime }}u_{j}^{{+}^{\prime }}}\delta_{ik}+\bar{u_{i}^{{+}^{\prime }}u_{j}^{{+}^{\prime }}u_{k}^{{+}^{\prime }}}\right)}}+\frac{\beta }{Re_{\tau }}\frac{\partial^{2}\bar{u_{i}^{{+}^{\prime }}u_{j}^{{+}^{\prime }}}}{\partial x_{k}^{*2}}-\underset{\varepsilon_{ij}}{\underbrace{2\frac{\beta }{Re_{\tau }}\bar{\frac{\partial u_{i}^{{+}^{\prime }}}{\partial x_{k}^{*}}\frac{\partial u_{j}^{{+}^{\prime }}}{\partial x_{k}^{*}}}}}\\ +\underset{E_{ij}}{\underbrace{\frac{1-\beta }{We_{\tau }}\frac{\partial }{\partial x_{k}^{*}}\left(\bar{u_{i}^{{+}^{\prime }}c_{jk}^{{+}^{\prime }}}+\bar{u_{j}^{{+}^{\prime }}c_{ik}^{{+}^{\prime }}}\right)}}\underset{F_{ij}}{\underbrace{-\frac{1-\beta }{We_{\tau }}\bar{c_{ik}^{{+}^{\prime }}\frac{\partial u_{j}^{{+}^{\prime }}}{\partial x_{k}^{*}}+c_{jk}^{{+}^{\prime }}\frac{\partial u_{i}^{{+}^{\prime }}}{\partial x_{k}^{*}}}}} \end{array}$$

All these Equations contain high-order moments $A_{ij}$, $B_{ij}$, $C_{ij}$, $\phi_{ij}$, $T_{ij}$, $\varepsilon_{ij}$, $E_{ij}$, $F_{ij}$ and $\bar{u_{i}^{{+}^{\prime }}u_{j}^{{+}^{\prime }}}$. They are new unknowns. Apparently, the number of unknowns exceeds the number of Equations, so that all the high-order unknowns need to be modeled as functions of time-average variables.

## 3. Modeling of High-Order Moments of Fluctuations

Different from Newtonian turbulent flow, the above high-order moments of drag-reducing flow are all related to viscoelasticity. The terms directly related to viscoelasticity ($A_{ij}$, $B_{ij}$, $C_{ij}$, $E_{ij}$, $F_{ij}$) do not appear in the turbulent models of Newtonian flow, such that they are modeled for the first time. The other terms ($\phi_{ij}$, $T_{ij}$, $\varepsilon_{ij}$) have implicit and complex relations with viscoelasticity, such that the modeling manner of these terms uses the same way of Newtonian flow. The modeling process is as follows.

### 3.1. High-Order Moments Directly Related to Viscoelasticity

The additional turbulent diffusion terms introduced by viscoelasticity ($A_{ij}$, $E_{ij}$) and the correlation of conformation tensor $B_{ij}$ are much smaller than turbulent diffusion. Thus, they can be neglected. To conform the physical process and consider the easy use of the model, $C_{ij}$ is assumed to have relations with Reynolds stress, mean strain rate, and so on. According to the positive definiteness of Reynolds stress and nonpositive definiteness of mean strain rate, the dimensional form of $C_{ij}$ can be expressed as:

(12) $${C_{ij}}^{\dim}=\varphi_{1}f_{w}\lambda \frac{\rho }{T_{t}}\bar{{u_{i}}^{\prime }{u_{j}}^{\prime }}+\varphi_{2}\frac{1}{T_{t}}\left(\bar{c_{ij}}-\eta \delta_{ij}\right)$$

where ${C_{ij}}^{\dim}=C_{ij}\eta u_{\tau }/h$, $f_{w}=1-\exp\left(-y^{+}/100\right)$, $y^{+}$ is the dimensionless distance to the lower wall of the channel, $T_{t}$ ($={\left(\nu /\varepsilon \right)}^{1/2}$) is Kolmogorov time scale calculated from Kolmogorov length scale (${\left(\nu^{3}/\varepsilon \right)}^{1/4}$) and Kolmogorov velocity scale (${\left(\varepsilon \nu \right)}^{1/4}$). ν (=μ/ρ) is kinetic viscosity of fluid.

Equation (8) is nondimensionalized using $\varepsilon^{+}=\varepsilon /\left(u_{\tau }^{3}/h\right)$, fluid density, viscosity, and frictional velocity and combined with the definitions of $Re_{\tau }$, $We_{\tau }$, and β. The model of $C_{ij}$ is as follows:

(13) $$C_{ij}=\varphi_{1}f_{w}\frac{We_{\tau }}{\left(1-\beta \right)\sqrt{\beta /\left(Re_{\tau }\varepsilon^{+}\right)}}\bar{u_{i}^{{+}^{\prime }}u_{j}^{{+}^{\prime }}}+\varphi_{2}\frac{1}{\sqrt{\beta /\left(Re_{\tau }\varepsilon^{+}\right)}}\left(\bar{c_{ij}^{+}}-\delta_{ij}\right))$$

where $\varphi_{1}=0.05$, $\varphi_{2}=-0.01$. From observations, $F_{ij}$ and $C_{ij}$ have the following simple relation:

(14) $$F_{ij}=-\frac{1-\beta }{We_{\tau }}C_{ij}$$

### 3.2. High-Order Moments Indirectly Related to Viscoelasticity

For turbulent diffusion terms $T_{ij}$, Daly and Harlow’s model [26] is applied:

(15) $$T_{ij}=\frac{\partial }{\partial x_{k}^{*}}\left(C_{s}\frac{k^{+}}{\varepsilon^{+}}\bar{u_{k}^{{+}^{\prime }}u_{l}^{{+}^{\prime }}}\frac{\partial \bar{u_{i}^{{+}^{\prime }}u_{j}^{{+}^{\prime }}}}{\partial x_{l}^{*}}\right)$$

where $k^{+}$ and $\varepsilon^{+}$ are dimensionless turbulent kinetic energy and energy dissipation rate. The model parameter $C_{s}$ is 0.22.

Turbulent dissipation term $\varepsilon_{ij}$ and redistribution term $\phi_{ij}$ are modeled together:

(16) $$-\varepsilon_{ij}+\phi_{ij}=-\frac{2}{3}\delta_{ij}\varepsilon^{+}+\phi_{\left(1\right)ij}+\phi_{\left(2\right)ij}$$

where $\phi_{\left(1\right)ij}=-C_{1}\frac{\varepsilon^{+}}{k^{+}}\left(\bar{u_{i}^{{+}^{\prime }}u_{j}^{{+}^{\prime }}}-\frac{2}{3}k^{+}\delta_{ij}\right)$, $\phi_{\left(2\right)ij}=-C_{2}\left(P_{ij}-\frac{2}{3}\delta_{ij}P\right)-C_{3}\left(D_{ij}-\frac{2}{3}\delta_{ij}P\right)-C_{4}k^{+}\left(\frac{\partial \bar{u_{i}^{+}}}{\partial x_{j}^{*}}+\frac{\partial \bar{u_{j}^{+}}}{\partial x_{i}^{*}}\right)$, $P=P_{kk}/2$, $D_{ij}=-\left(\bar{u_{j}^{{+}^{\prime }}u_{k}^{{+}^{\prime }}}\frac{\partial \bar{u_{k}^{+}}}{\partial x_{i}^{*}}+\bar{u_{i}^{{+}^{\prime }}u_{k}^{{+}^{\prime }}}\frac{\partial \bar{u_{k}^{+}}}{\partial x_{j}^{*}}\right)$. Shima [27] gave the expressions of coefficients:

(17) $$C_{1}=1+2.45A_{2}^{1/4}A^{3/4}\left\{1-\exp\left[-{\left(7A\right)}^{2}\right]\right\}\left\{1-\exp\left[-{\left(R_{T}/60\right)}^{2}\right]\right\}$$

(18) $$C_{2}=0.7A$$

(19) $$C_{3}=0.3A^{1/2}$$

(20) $$C_{4}=0.65A\left(0.23C_{1}+C_{2}-1\right)+1.3A_{2}^{1/4}C_{3}$$

where $A=1-9A_{2}/8+9A_{3}/8$, $A_{2}=a_{ij}a_{ji}$, $A_{3}=a_{ij}a_{jk}a_{ki}$, $a_{ij}=\bar{u_{i}^{{+}^{\prime }}u_{j}^{{+}^{\prime }}}/k^{+}-2\delta_{ij}/3$, $R_{T}=k^{+2}/\left(\varepsilon^{+}\beta /Re_{\tau }\right)$.

The above modeling introduces two new unknowns, $k^{+}$ and $\varepsilon^{+}$, which need be modeled. $k^{+}$ can be solved from the definition of turbulent kinetic energy ($k^{+}=\bar{u_{i}^{{+}^{\prime }}u_{i}^{{+}^{\prime }}}/2$), where $\bar{u_{i}^{{+}^{\prime }}u_{i}^{{+}^{\prime }}}$ can be solved from the Reynolds stress transport Equation (Equation (11)). $\varepsilon^{+}$ can be solved from Shima’s model [27]:

(21) $$\frac{\partial \varepsilon^{+}}{\partial t^{*}}+\bar{u_{k}^{+}}\frac{\partial \varepsilon^{+}}{\partial x_{k}^{*}}=C_{\varepsilon 1}\frac{\varepsilon^{+}}{k^{+}}P-C_{\varepsilon 2}\frac{\varepsilon^{+}{\tilde{\varepsilon }}^{+}}{k^{+}}+\frac{\partial }{\partial x_{k}^{*}}\left(C_{\varepsilon }\frac{k^{+}}{\varepsilon^{+}}\bar{u_{k}^{{+}^{\prime }}u_{l}^{{+}^{\prime }}}\frac{\partial \varepsilon^{+}}{\partial x_{l}^{*}}+\frac{\beta }{Re_{\tau }}\frac{\partial \varepsilon^{+}}{\partial x_{k}^{*}}\right)$$

where ${\tilde{\varepsilon }}^{+}=\varepsilon^{+}-2\frac{\beta }{Re_{\tau }}{\left[\frac{\partial {\left(k^{+}\right)}^{1/2}}{\partial x_{l}^{*}}\right]}^{2}$, $C_{\varepsilon 1}=1.44+\beta_{1}+\beta_{2}$, $C_{\varepsilon }=0.15$, $C_{\varepsilon 2}=1.92$,

$\beta_{1}=0.25A\min\left(\lambda^{\prime }/2.5-1,0\right)-1.4A\min\left(P/\varepsilon^{+}-1,0\right)$, $\beta_{2}=1.0A{\lambda^{\prime }}^{2}\max\left(\lambda^{\prime }/2.5-1,0\right)$,

$\lambda^{\prime }=\min\left(\lambda^{*},4\right)$, $\lambda^{*}={\left[\frac{\partial }{\partial x_{i}^{*}}\left(\frac{k^{+3/2}}{\varepsilon^{+}}\right)\frac{\partial }{\partial x_{i}^{*}}\left(\frac{k^{+3/2}}{\varepsilon^{+}}\right)\right]}^{1/2}$.

All the high-order moments are modeled to be the functions of time-average variables. Equations (8)–(17) combined with Equations (4), (5), and (7) compose a Reynolds stress model.

## 4. Results and Discussion

The above Reynolds stress model was used to simulate the fully developed viscoelastic drag-reducing channel flow. The computational domain is shown in Figure 1. Periodic boundary conditions were imposed in both the streamwise (*x*-) and spanwise (*z*-) directions, while nonslip boundary conditions were adopted for the top and bottom walls. Computational parameters were: $Re_{\tau }=150$, $We_{\tau }=10$, α=0.001, β=0.8.

The numerical method of DNS is a fractional step method. Adams–Bashforth scheme was used to ensure the second-order accuracy of velocity. An implicit scheme was used for the pressure term. Staggered grid was applied to avoid unphysical oscillations of pressure. A second-order finite difference scheme was used for spatial discretization. Uniform mesh was used in the *x* and *z* directions due to the periodic boundary condition. To capture small eddies near the walls, nonuniform mesh was used in the *y* direction. Grid number was 64 × 64 × 64. Drag reduction is defined as:

(22) $$DR\%=\frac{C_{fDean}-C_{f}}{C_{fDean}}\times 100\%$$

where *C_f_* is the calculated friction factor, *C_fDean_* is evaluated by Dean’s correlation [28], *DR%* is the drag reduction.

Mean streamwise velocity, drag reduction, Reynolds stress, and fluctuation intensity were obtained and compared with the results of DNS to validate the model. Bulk mean variables are listed in Table 1. The results obtained by the turbulent model (RSM) agree well with the DNS results. The relative deviations of mean streamwise velocity, Reynolds number, frictional factor, and drag reduction were 0.57%, 0.57%, 1.27%, and 9.3%. Thus, the prediction of the bulk mean variables by RSM is accurate.

**Figure 2:**
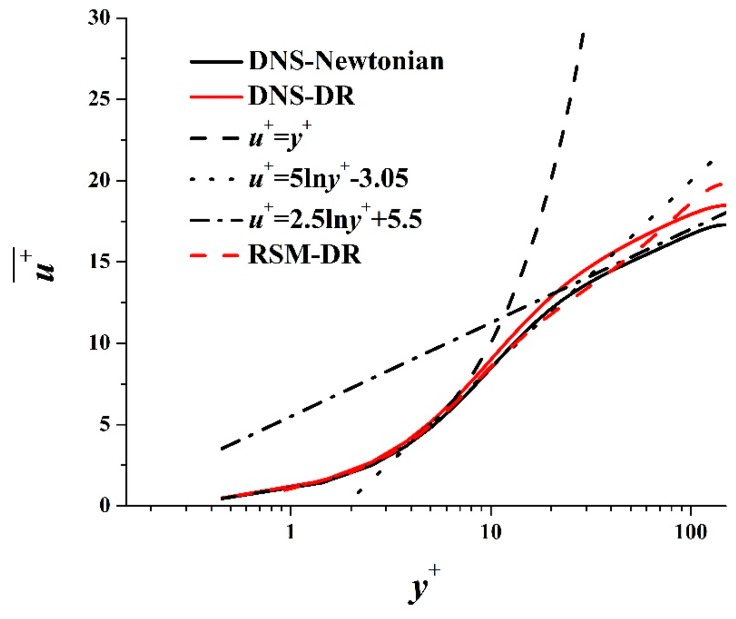
Time-average streamwise velocity profiles.

**Figure 3:**
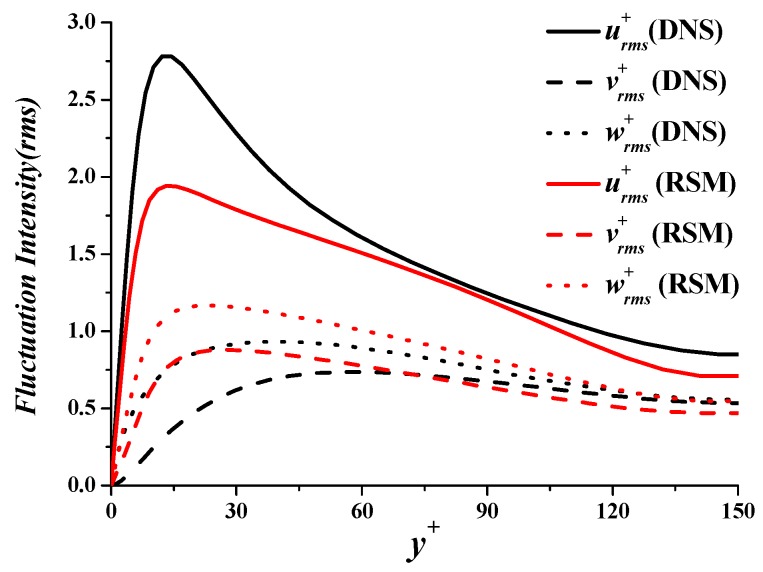
Fluctuation intensities.

Figure 2 is the comparison of time-average streamwise velocity profiles between RSM and DNS. The DNS results for Newtonian turbulent flow agree well with the typical distribution of viscous sublayer ($u^{+}=y^{+}$), buffer layer ($u^{+}=5\ln y^{+}-3.05$), and turbulent core region ($u^{+}=2.5\ln y^{+}+5.5$) [29], showing that the fluid has achieved fully developed turbulent flow. The time-average velocity of RSM coincides well with DNS in the viscous sublayer, is lower than DNS in the buffer layer, and is higher than DNS in the turbulent core region. The mean deviation is only 9.1%, which is much lower than previous turbulent models (as high as 30–50% or more). This indicates the established model can predict the time-average streamwise velocity profile accurately. Fluctuation intensities are compared in Figure 3. Predicted $v_{rms}^{+}$ and $w_{rms}^{+}$ are larger than DNS, while $u_{rms}^{+}$ is smaller than DNS. The peak value positions of $u_{rms}^{+}$ $v_{rms}^{+}$, and $w_{rms}^{+}$ of RSM are basically the same as DNS.

**Figure 4:**
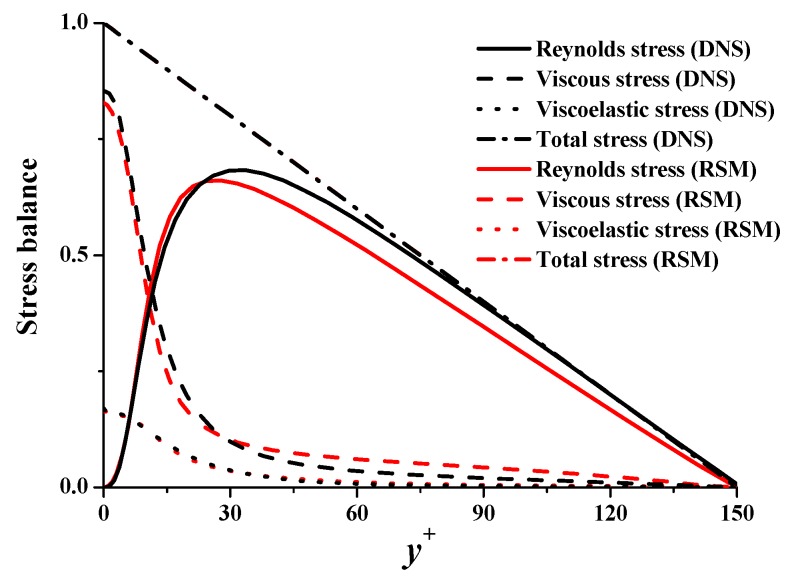
Stress balance.

Stress balance of Reynolds stress, viscous stress, and viscoelastic stress is shown in Figure 4. Prediction results of the three stresses agree well with those of DNS. Predicted Reynolds stress is smaller than DNS. Predicted viscoelastic stress is almost the same as that of DNS.

**Figure 5:**
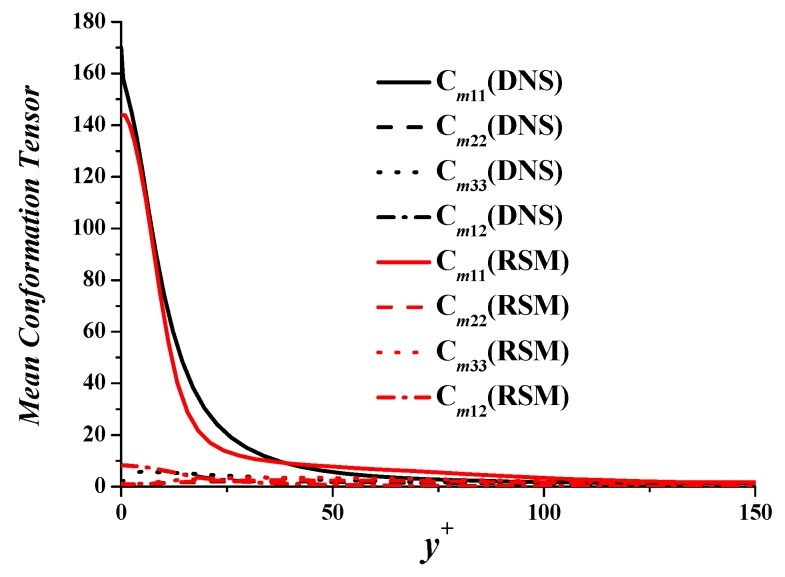
Mean conformation tensor.

$C_{mij}$ represents the mean conformation tensor $\bar{c_{ij}^{+}}$. The four main components of the conformation tensor, with subscripts 1, 2, 3 representing streamwise, wall-normal, and spanwise directions, are compared in Figure 5. It shows that the four components agree well with DNS. The peak value of $C_{m11}$ is 15% lower than the value in DNS. $C_{m12}$ of RSM coincides well with DNS. It determines the value of viscoelastic stress, such that the deviation of viscoelastic stress is small in Figure 4.

**Figure 6:**
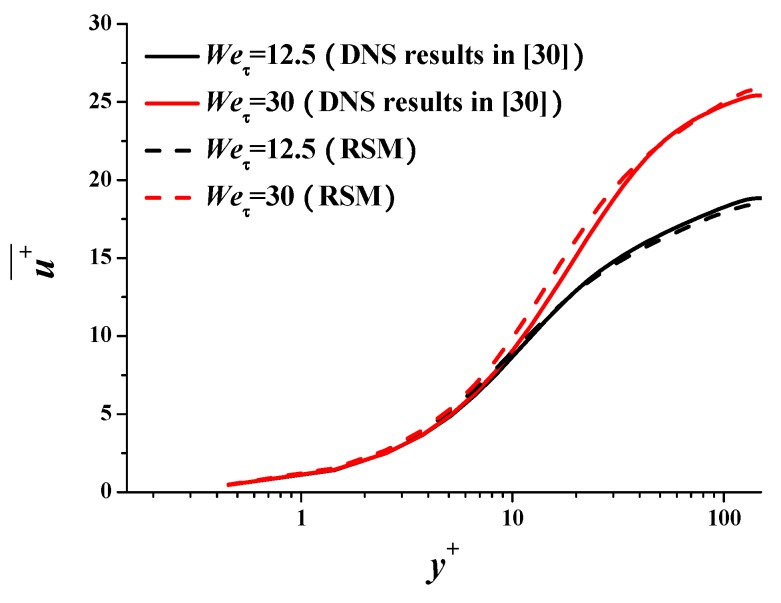
Mean velocity profile comparison with literature.

**Figure 7:**
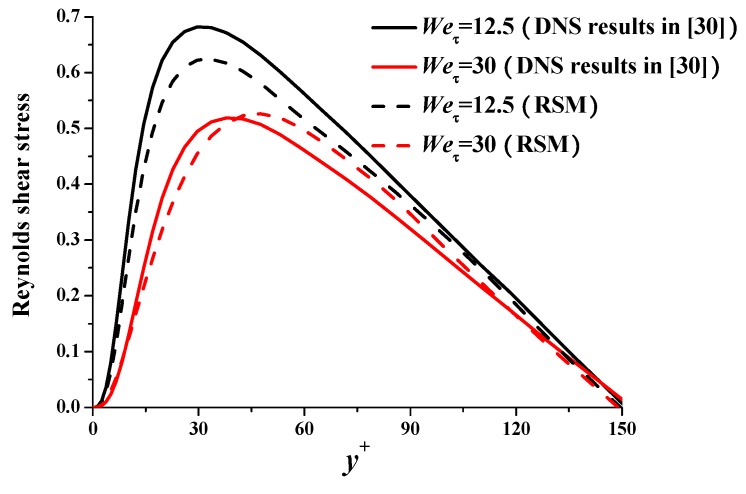
Reynolds shear stress comparison with literature.

The RSM for the above case is basically verified. We further verify the RSM using the DNS results in literature [30] at different Weissenberg numbers. Table 2 shows the bulk mean variables such as drag reduction. It is apparent that the RSM can also simulate the drag-reducing flow accurately at *We*_τ_ = 12.5 and 30, which correspond to low and medium drag reduction, respectively. Figure 6 and Figure 7 show that the critical features (e.g., average velocity profile and Reynolds stress distribution) can be simulated by the RSM in satisfied precision.

RSM only concerns time-average variables so that it does not need to resolve the small eddies near the walls like DNS. The complex and time-consuming numerical methods, such as numerous iterations of pressure, can be avoided. Therefore, the computational time of RSM can be much smaller than that of DNS. Table 3 verifies that RSM can largely save the computational time because the acceleration ratio of computational time of DNS and RSM is as high as 120,960.

To further examine the precision of the RSM at higher Reynolds numbers, it is compared with the typical DNS results made by Housiadas et al. [31] in Table 4. The drag reduction of the RSM agrees well with the DNS data in low (*Re*_τ_ = 125) and medium (*Re*_τ_ = 180) Reynolds numbers. The deviation becomes larger at higher Reynolds number (*Re*_τ_ = 395). The model was also used to predict the effect of Weissenberg number at the higher Reynolds number (Table 5). Drag reduction increases with increasing *We*_τ_ and tends to a stable value about more than 40%.

## 5. Conclusions

Through the discussions, it is apparent that the newly established Reynolds stress turbulent model can predict bulk mean velocity and drag reduction accurately. The predictions of stresses and mean conformation tensor are also good. The time-average streamwise velocity outside the viscous sublayer is quite different from that in DNS. Streamwise fluctuation intensity is much smaller than that in DNS. This is probably because the modeling of the redistribution term $\phi_{ij}$ is the same as that for Newtonian fluid. This term may affect viscoelasticity and pressure in a complex way, which needs further study in future to find a more accurate modeling manner. The acceleration ratio is as high as 120,960, showing the extremely high speed of the new turbulent model of polymeric drag-reducing flow. The Reynolds stress model appears to have good accuracy at lower Reynolds numbers compared with typical DNS results. The model predictions significantly worsen as the Reynolds number and Weissenberg number increase, limiting the applicability of the model up to modest levels of drag reduction. This suggests (in association with the other limitations already stated before) that there is room for model improvement in future work.

## Figures and Tables

**Table 1:** Comparison of bulk mean variables.

	$\bar{u_{m}^{+}}$	*Re*_m_	*C*_f_	*DR*%
Direct numerical simulation (DNS)	15.87	4762	0.0079	9.7%
Reynolds stress model (RSM)	15.96	4789	0.0078	10.6%

**Table 2:** Comparison of bulk mean variables with literature [30].

	$\bar{u_{m}^{+}}$	*Re*_m_	*C*_f_	*DR*%
DNS (*We*_τ_ = 12.5) [30]	16.13	4838	0.00769	12.1%
RSM (*We*_τ_ = 12.5)	16.10	4829	0.00772	11.9%
DNS (*We*_τ_ = 30) [30]	20.6	6180	0.00471	42.8%
RSM (*We*_τ_ = 30)	21.2	6373	0.00443	45.8%

**Table 3:** Computational time.

DNS	RSM	Acceleration Ratio
604,800s	5s	120,960

**Table 4:** Drag reduction (*DR*%) comparison with Housiadas’ results [31] at different *Re*_τ_.

Case	DNS	RSM	Deviation
*Re*_τ_ = 125, *We*_τ_ = 50, *β* = 0.9	29.8%	29.2%	2.0%
*Re*_τ_ = 180, *We*_τ_ = 50, *β* = 0.9	29.9%	28.6%	4.3%
*Re*_τ_ = 395, *We*_τ_ = 50, *β* = 0.9	29.6%	25.7%	13.2%

**Table 5:** *DR*% at different *We*_τ_ of the RSM for high *Re*_τ_.

Case	*DR*%
*Re*_τ_ = 395, *We*_τ_ = 25, *β* = 0.9	24.7%
*Re*_τ_ = 395, *We*_τ_ = 50, *β* = 0.9	25.7%
*Re*_τ_ = 395, *We*_τ_ = 100, *β* = 0.9	28.0%
*Re*_τ_ = 395, *We*_τ_ = 150, *β* = 0.9	33.8%
*Re*_τ_ = 395, *We*_τ_ = 200, *β* = 0.9	41.1%
*Re*_τ_ = 395, *We*_τ_ = 250, *β* = 0.9	44.4%
